# Divergent seasonal patterns of influenza types A and B across latitude gradient in Tropical Asia

**DOI:** 10.1111/irv.12372

**Published:** 2016-02-05

**Authors:** Siddhartha Saha, Mandeep Chadha, Yuelong Shu, Group of Asian Researchers on Influenza (GARI), Wang Lijie, Malinee Chittaganpitch, Sunthareeya Waicharoen, Kim A. Lindblade, Vongphrachanh Phengta, Darouny Phonekeo, Andrew Corwin, Sok Touch, Philippe Buchy, Raymond Lin, Constance Low, Chong Chee Kheong, Apandi bin Yusof, Amado Tandoc, Vito Roque, Vina Arguelles, Fatimah S. Dawood, Ann Moen, Marc‐Alain Widdowson, Nancy J. Cox, Renu B Lal

**Affiliations:** ^1^ US CDC‐IndiaNew DelhiIndia; ^2^ National Institute of VirologyPuneIndia; ^3^ Chinese National Influenza CenterBeijingChina

**Keywords:** Human influenza, influenza in tropics, influenza seasonality, influenza surveillance

## Abstract

**Introduction:**

Influenza circulation in tropics and subtropics reveals a complex seasonal pattern with year‐round circulation in some areas and biannual peaks in others.

**Methods:**

We analyzed influenza surveillance data from nine countries around southern and southeastern Asia spanning latitudinal gradient from equatorial to temperate zones to further characterize influenza type‐specific seasonality in the region. We calculated proportion of positives by month out of positives during that year and adjust for variation in samples tested and positivity in these countries.

**Results:**

Influenza A epidemics were identified between November and March during winters in areas lying above 30°N latitude, during monsoon months of June–November in areas between 10° and 30°N latitude, and no specific seasonality for influenza A virus circulation in areas lying closer to the equator. Influenza B circulation coincided with influenza A circulation in areas lying above 30°N latitude; however, in areas south of 30°N Asia, influenza B circulated year round at 3–8% of annual influenza B positives during most months with less pronounced peaks during post‐monsoon period.

**Conclusion:**

Even though influenza B circulates round the year in most areas of the tropical regions of southern and southeastern Asia, the most appropriate time for influenza vaccination using the most recent WHO recommended vaccine would be prior to the monsoon season conferring protection against influenza A and B peaks.

## Introduction

Seasonal influenza viruses typically circulate between November and March in the Northern Hemisphere and between April and September in the Southern Hemisphere.^1^, ^2^ While influenza surveillance data have been used to inform vaccination strategies in temperate regions, historically data from countries in the tropics and subtropics have been limited.^3^, ^4^ With the strengthening of influenza surveillance in recent years supported by the use of reverse transcription–polymerase chain reaction, more data on influenza activity in the tropics and subtropics are available, showing a complex seasonal pattern with year‐round circulation in some areas and biannual peaks in others.^4^ The more diffuse patterns of influenza activity further complicate vaccination recommendations, specifically timing of vaccination, for countries in the tropics.^5^ Recent surveillance data from tropical regions of Asia have revealed a seasonal pattern characterized by year‐round low‐level influenza virus circulation with peaks occurring during the monsoon (rainy) season.^6^ The distinct pattern has important implications for influenza vaccine timing in this region.^4^, ^6^, ^7^

Availability of surveillance data for influenza subtype/lineage from tropical regions of Asia, together with recent efforts to introduce quadrivalent influenza vaccines, prompted us to undertake in‐depth analysis of data from nine countries around southern and southeastern Asia to further characterize influenza type‐ and subtype‐specific seasonality and associated latitude gradient in this region.

## Methods

### Study design

Monthly surveillance data from 2007 to 2013 (*N* = 1 586 757 specimens) collected as part of the World Health Organization's Global Influenza Surveillance and Response System (GISRS) were obtained directly from eight countries in the Asian region, while data for Japan were accessed from the FluNet server [http://www.who.int/influenza/gisrs_laboratory/flunet/en/] (Table 1). Nasopharyngeal and/or throat swabs from patients presenting with influenza‐like illness (ILI) or hospitalized with severe acute respiratory infection (SARI) at sentinel surveillance sites were tested for influenza viruses either by virus isolation in Madin–Darby canine kidney (MDCK) cells or by real‐time reverse transcription–polymerase chain reaction (RT‐PCR) using standard protocols 8 and considered positive if detected by either method. Influenza virus typing and subtyping were carried out using United States Centers for Disease Control and Prevention (CDC) protocols.^9^ Influenza B lineage testing was carried out for a subset of influenza‐positive specimens and increased after 2009.

**Table 1:** Specimens tested and influenza positivity results in the nine study countries of Asia

Country/Regions	Total samples			Influenza A				Influenza B			
	Specimens tested (#)	Influenza positive (#)	Sample Positivity (%)	Positive (#)	Proportion Positive (%)	Epidemic months^a^	Season	Positive (#)	Proportion Positive (%)	Epidemic months^a^	Season
Japan^b^	NA	76 374		66 373	86·9	December–March	Winter	10 001	13·1	December–April	Winter
North China	547 154	77 824	14·2	55 889	71·8	November–March	Winter	21 935	28·2	December–March	Winter
South China	820 595	141 771	17·3	102 612	72·4	July–September and January–March	Monsoon–Winter	39 159	27·6	December–April	Winter
India	29 319	3471	11·8	2155	62·1	June–August	Monsoon	1313	37·8	August–December	Monsoon–Winter
Lao PDR^c^	5701	1026	18·0	684	66·7	September–December	Monsoon	342	33·3	July–January	Monsoon–Winter
Thailand	25 826	5276	20·4	3408	64·6	July–November	Monsoon	1868	35·4	August–February	Monsoon–Winter
Cambodia	13 346	2103	15·8	1226	58·3	July–October	Monsoon	877	41·7	September–January	Monsoon–Winter
Philippines	73 629	12 698	17·2	10 635	83·8	July–October	Monsoon	2063	16·2	September–December	Monsoon–Winter
Malaysia	11 285	805	7·1	577	71·7	–		233	28·9	April–May, August–October	Monsoon–Winter
Singapore	59 902	14 750	24·6	12 310	83·5	May–June	Monsoon	2440	16·5	January–February, April–May, November–December	Monsoon–Winter
Total^d^	1 586 757	259 724	16·4	189 496	73·0	–		70 230	27·0	–	

a Defined as 2 or more consecutive months with monthly proportion of annual positive samples above 8·3%.

b Data from FluNet.

c Data for 2008–2013.

d Excluding data from Japan.

### Data analysis

Data were analyzed by country, except for China which was analyzed by northern and southern China (Appendix 2: List of provinces). We used monthly instead of weekly surveillance data as we felt that it is more practicable for policy guidance for influenza seasonality and vaccination timing especially at the regional level. We calculated proportion of influenza A‐ and influenza B‐positive samples separately in a month out of total influenza A‐ and total B‐positive samples, respectively, in a given year to analyze the monthly pattern. This allowed us to adjust for inter‐ and intracountry variations in terms of samples tested and sample positivity. The epidemic period for each influenza type was defined as two or more consecutive months with monthly proportion of annual positive samples above 8·3% for that type, that is the average percentage of cases detected per month (100% divided by 12 months). The peak month was defined as the month with highest average proportion of annual positives. We plotted the seasonal patterns of areas/countries using Epi‐Map to identify the latitudinal gradient.

### Ethical review

The study used aggregated data from each country collected for influenza surveillance as part of FluNet.

## Results

During 2007–2013, results from testing of 1 586 757 specimens were available for analysis from eight countries (data for Japan – not available) (Table 1). A total of 336 098 influenza‐positive samples were detected including Japan (*n* = 76 734), northern China (*n* = 77 824), southern China (*n* = 141 771), India (*n* = 3471), Lao PDR (*n* = 1026), Thailand (*n* = 5276), Cambodia (*n* = 2103), the Philippines (*n* = 12 698), Malaysia (*n* = 805) and Singapore (*n* = 14 750). The proportion of specimens testing positive for influenza viruses ranged from 7 to 25%. Although influenza A viruses accounted for a larger proportion of all influenza‐positive specimens during most years, influenza B viruses accounted for >50% of influenza positives in 2010–2011 and 2012–2013 (data not shown).

**Figure 1:**
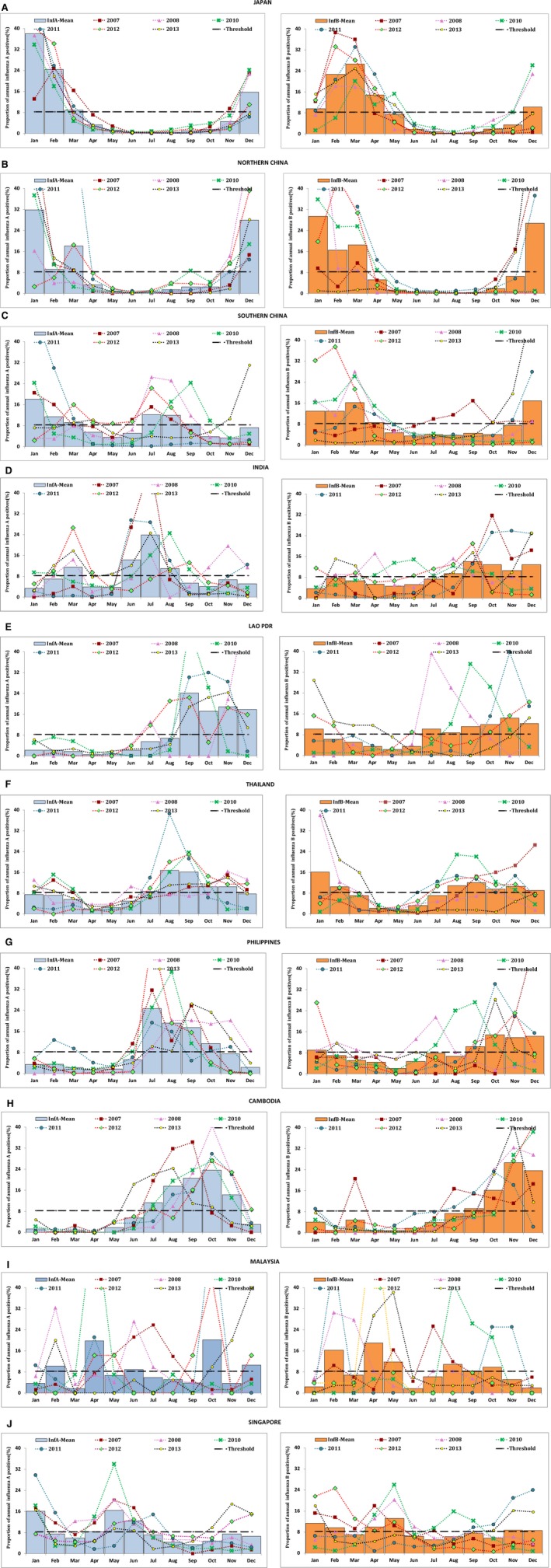
Consensus seasonality of influenza A and B in the nine study countries of Asia (2007–2013 excluding 2009). Each panel graph is for each country/region, and bars denote mean of monthly type‐specific proportion of annual number of influenza type‐positive samples. The bluish‐gray bars indicate influenza A and orange bars indicate influenza B. The horizontal dashed line denotes threshold value of 8·3% of annual positive. Monthly pattern of influenza proportion positives in each year is shown by line graph; the data for 2009 were excluded because of the pandemic which did not follow the usual seasonality pattern.

**Figure 2:**
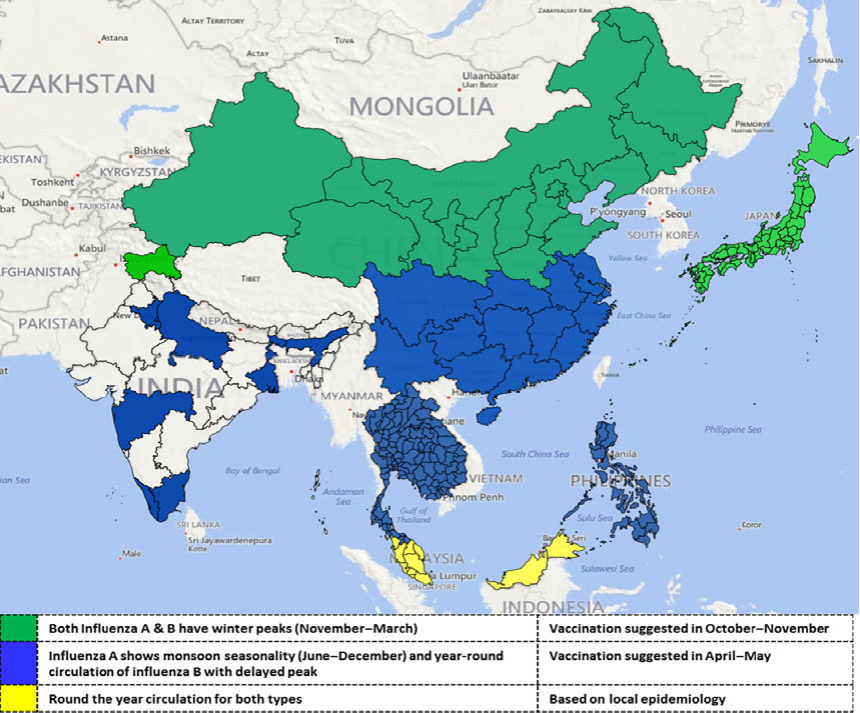
Influenza virus circulation patterns in the nine study countries of Asia, 2007–2013. Areas with winter peaks of influenza are indicated by green color, and areas with monsoon peaks of influenza are indicated by blue, and areas with year‐round circulation with no distinct peaks have been shaded yellow.

The study revealed three patterns of influenza A seasonality that coincided with the latitudinal position of countries (Figures 1 and 2). First, areas lying above 30°N latitude (Japan and northern China) were characterized by influenza A epidemics during winter months of November–March. Second pattern was observed in areas between 10° and 30°N latitude (southern China, India, Thailand, Lao PDR, Cambodia and the Philippines) that have influenza A epidemics during monsoon months of June–December although some areas (southern China, India, Thailand) showed 3–8% of annual influenza A positives even during non‐epidemic months. Third pattern was seen in areas lying close to the equator approx. 0–10°N latitude (Malaysia and Singapore) displaying no specific seasonality in influenza A virus circulation (Figures 1 and 2). In contrast, there were two patterns to influenza B circulation in this region: a distinct seasonality coinciding with influenza A circulation in areas lying north of 30°N latitude (Japan and northern China); while in areas south of 30° N (except Cambodia) year‐round circulation with 3–8% of annual influenza B positives during most months with less pronounced and lagged peaks after influenza A peaks occurring between September and February.

**Figure 3:**
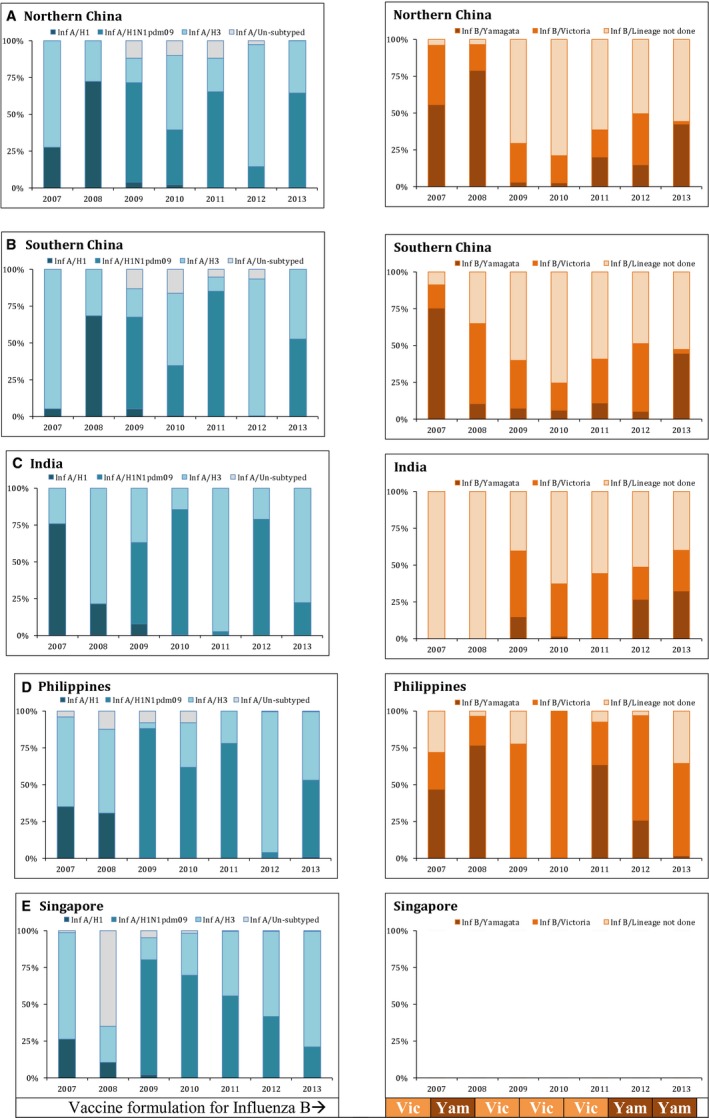
Subtype variation in selected study countries in the Asian region (2007–2013). Each panel graph is for each country and influenza type, and stacked bars denote the circulating subtypes during the year.

The predominant circulating influenza A subtype and B lineage varied by year. The predominant influenza A subtype was A/H1N1pdm09 during 2009–2011 and A/H3N2 in 2012, whereas both subtypes were codominant in 2013 (Figure 3). Similarly data on influenza B lineage revealed B/Yamagata‐lineage viruses predominated during 2007–2008, and B/Victoria‐lineage during 2009–2010 and cocirculation of both lineages during 2012. B/Yamagata‐lineage again became the predominant strain in 2013. Thus, both influenza B lineage viruses in varying proportions circulated in most years across most countries (Figure 3).

## Discussion

This study indicated that in the tropical regions of southern Asia, influenza A viruses cause seasonal epidemics typically during monsoon months, whereas influenza B viruses circulate year round with smaller peaks during the post‐monsoon months. In contrast, in the temperate region, both influenza A and B viruses peak during the winter months, similar to other countries in temperate regions of the Northern Hemisphere and Southern Hemisphere.^1^, ^5^ The subnational data from India and China revealed that areas at approximately ≥30°N latitude have winter peaks of both influenza A and B virus circulation, whereas areas in these countries that lie below <30°N latitude, influenza A peaks occur primarily during the monsoon season with influenza B circulation year round with smaller peaks during post‐monsoon months.^10^, ^11^, ^12^While reason for discrepant seasonality in circulation of influenza A and B viruses in tropical Asia is not clear, it is plausible that either environmental or other factors in tropical regions play a role in sustained circulation of influenza B viruses throughout the year. Additional high‐quality surveillance along with systematically collected data on climatic and environmental variables from tropical countries would allow further evaluation of relationship between these factors and influenza seasonality.^11^, ^13^, ^14^

World Health Organization makes biannual recommendations for the composition of seasonal influenza vaccines for Northern Hemisphere and Southern Hemisphere.^15^ Until recently, the trivalent vaccine included influenza A(H1N1), A(H3N2), and B strains with only one of the two B lineages represented; however, a quadrivalent vaccine comprising of two each of influenza A and B lineages is being licensed in some countries. The data presented here showing cocirculation of both lineage of influenza B further lend support to possible use of quadrivalent vaccine in tropical Asian countries, if available.

We previously suggested that the most appropriate time for influenza vaccination in the tropical regions of southern and southeastern Asia would be during April–May prior to the monsoon season instead of October–November as is practiced in temperate regions of Northern Hemisphere,^6^ and similar recommendations can be made from current data. Even though round the year circulation of influenza B complicates the timing of vaccination in areas south of 30°N, the pre‐monsoon vaccination time should confer protection against influenza B peak season. However, the vaccine using Northern Hemisphere strain of the coming season will not be available in April–May, so countries may need to consider using the Southern Hemisphere strain which will have the most recent WHO‐recommended strains.

The data reported here have limitations; first, the surveillance systems in these countries differ in terms of population coverage and data collection methods. Additionally, we used monthly data, which precluded some of the statistical analysis possible for weekly data. Secondly, we used aggregated data for China and India both of which have vast latitudinal expanse, and the surveillance is relatively sparse in some parts of these countries and so may not have completely captured the complexity of influenza seasonality across the latitude gradient,^11^, ^12^ which we tried to partly address by analyzing China data by epidemiologically distinct northern and southern regions. Lastly, influenza B lineage data were restricted to some years and only subsamples in some countries in the region, further limiting our ability to in‐depth analysis. Nevertheless, we clearly show cocirculation of both influenza B lineages throughout the year in the tropical regions of Asia. Despite these limitations, we believe that our data provide first comprehensive analysis of both influenza type and subtype circulation across a wide latitude gradient in tropical Asia.

In summary, we provide evidence that while both influenza A and B viruses cause seasonal epidemics during the winter in temperate regions of Asia; in contrast, influenza A shows seasonal peak during monsoon with influenza B showing a lagged peak during the post‐monsoon/autumn season in tropical regions of Asia. Most importantly, cocirculation of both subtypes of influenza A and both lineages of influenza B was observed regardless of temperate or tropical climate.

## Funding

This study used the available influenza surveillance data collected as part of the World Health Organization's Global Influenza Surveillance and Response System (GISRS). The surveillance in most of these countries were supported by U. S. Centers for Disease Control and Prevention (CDC) through cooperative agreements. No separate funding was received for this study.

## Conflict of interest

None of the authors except PB have declared any conflict of interest. PB is on sabbatical leave from Pasteur Institute, Phnom Penh, Cambodia and currently employed by GSK Vaccines in Asia Pacific region. The opinions expressed by authors contributing to this article do not necessarily reflect the opinions of the U.S. Centers for Disease Control and Prevention, or the authors' affiliated institutions.

## Appendix 1

Wang Lijie^c^, Malinee Chittaganpitch^d^, Sunthareeya Waicharoen^d^, Kim A. Lindblade^f^ Vongphrachanh Phengta^e^; Darouny Phonekeo^e^, Andrew Corwin^f^, Sok Touch^g^, Philippe Buchy^h,i^, Raymond Lin^j^, Constance Low^j^, Chong Chee Kheong^k^, Apandi bin Yusof^l^, Amado Tandoc III^m^, Vito Roque Jr^n^, Vina Arguelles^n^, Fatimah S. Dawood^f^, Ann Moen^f^, Marc‐Alain Widdowson^f^, Nancy J. Cox^f^ and Renu B Lal^a^

^a^US CDC‐India, New Delhi, India

^b^National Institute of Virology, Pune, India

^c^Chinese National Influenza Center, Beijing, China

^d^National Institute of Health, Nonthaburi, Thailand

^e^Ministry of Health, Vientiane, Lao People's Democratic  Republic

^f^Centers for Disease Control and Prevention, Atlanta, GA,  USA

^g^Ministry of Health, Phnom Penh, Cambodia

^h^Pasteur Institute, Phnom Penh, Cambodia

^i^GSK Vaccines, R&D, Gateway West, Singapore

^j^Ministry of Health, Singapore, Singapore

^k^Ministry of Health, Kuala Lumpur, Malaysia

^l^Institute of Medical Research, Kuala Lumpur, Malaysia

^m^Research Institute for Tropical Medicine, Alabang, Philip pines

^n^Department of Health, Manila, Philippines

## Appendix 2 Regional distribution of provinces of China.

**Table:**

Provinces in Northern China Region	Provinces in Southern China region
1. Beijing	1. Anhui
2. Gansu	2. Fujian
3. Hebei	3. Guangdong
4. Henan	4. Guangxi
5. Heilongjiang	5. Guizhou
6. Jilin	6. Hainan
7. Liaoning	7. Hubei
8. Inner Mongolia	8. Hunan
9. Ningxia	9. Jiangsu
10. Qinghai	10. Jiangxi
11. Shandong	11. Shanghai
12. Shanxi	12. Sichuan
13. Shaanxi	13. Yunnan
14. Tianjin	14. Zhejiang
15. Tibet	15. Chongqing
16. Xinjiang	
